# Dysentery

**Published:** 1828-12

**Authors:** 


### DYSENTERY.

*Cases of Chronic Dysentery, treated at the* Pennsylvania Hospital, *by* J. K. Mitchell, m.d.

The seamen whose cases follow were admitted on the afternoon of the 17th of May, 1828, with dysentery of five weeks' duration. They arrived a few days previous to their admission from a port in the West Indies, after a voyage of near four weeks, and attributed their sickness to the warm weather and bad water.

Case I.—Joseph Mitchell, aged thirty-eight years, seaman. Symptoms: Has had four slimy stools since morning; some pain in the bowels; vomited several times today; pulse seventy-five; tongue natural; skin moist.

Treatment: Ol. Ricini ℥i. statim. Pil. Cœrulæ grs. iij. at night.—Diet restricted to gum Arabic water and slippery elm tea.

18th, nine a.m.—Pulse, tongue, and skin natural; one stool since oil operated.

Directed grs. iij. Pil. Cœrulæ to be taken at bedtime, and to continue diet as yesterday.

19th, nine a.m.—Three stools in last twenty-four hours.

Diet: Tapioca and one cracker,* in addition to slippery elm tea and gum Arabic water.

20th, nine a.m.—Three stools, nearly natural; no pain.

Continue diet as yesterday.—Pil. Cœrulæ grs. iij. at bedtime.

21st, nine a.m.—One stool, natural.

Diet: Tapioca, boiled milk, crackers, and chicken soup, prepared by boiling the chicken in barley water.

24th.—Allowed the diet of the house. 26th.—Discharged well.

Case II.—Samuel Amee, aged twenty-five years, seaman. Symptoms: Has had six slimy stools since morning; tormina and tenesmus; pulse ninety, full and compressible; tongue red and rather glossy.

* A kind of small hard biscuit.

Treatment: Ol. Ricini ℥i. statim. Pil. Cœrulæ grs. iij. at night.—Diet restricted to slippery elm tree and gum Arabic water.

18th, nine A.M.—Two stools since oil operated, these more natural.

Continue diet.

Seven P.M.—Tongue less red; pulse seventy-seven; one stool since morning.

Continue same diet.—Pil. Cœrulæ grs. iij. at bedtime.

19th, nine A.M.—No stool since evening; tongue and pulse natural.

Diet: Tapioca and one cracker, in addition to slippery elm tea and gum water.

20th, nine A.M.—One stool, natural; no pain.

Diet same as yesterday.—Pil. Cœrulæ grs. iij. at night.

21st, nine A.M.—One stool.

Diet: Tapioca, boiled milk, cracker, and chicken soup.

24th.—Allowed the diet of the house. 26th.—Discharged well.

CASE III.—Andrew Amee, aged twenty years, seaman.

Symptoms: Has from fifteen to twenty stools in twenty-four hours, (has had fifty.) These are bloody and slimy, and accompanied with tormina and tenesmus; tongue red and glossy; pulse fifty-five; skin moist.

Treatment: Ol. Ricini ℥i. statim. Pil. Cœrulæ grs. iij. at night.—Diet restricted to slippery elm tea and gum Arabic water.

18th, nine A.M.—Five stools since oil operated; these less bloody and with less pain.—Continue diet.

Seven P.M.—Two stools since morning, more natural; pulse seventy-eight.

Pil. Cœrulæ gr. iij. at bedtime. Continue diet.

19th, nine A.M.—Three stools since evening; pulse eighty; tongue less red.

Diet: Tapioca and one cracker, in addition to slippery elm tea and gum water.

Seven P.M.—Not so well. Has had five bloody and slimy stools since morning, with more pain; pulse ninety-two; tongue red; skin hot and dry; pain in the head and bowels.

V.S. ℥xij. Diet restricted to slippery elm tea and gum water.

20th, nine A.M.—Better. Two stools, slimy but with very little blood, and attended with less pain; pulse, skin, and tongue more natural. Blood taken last evening was sizy.

Diet same as last night.

Seven P.M.—One stool, which was more natural and contained no blood.

Diet as before. Pil. Cœrulæ gr. iij. at bedtime.

21st, nine A.M.—One stool, almost natural; no pain.

Diet: Tapioca and one cracker, in addition to slippery elm tea and gum water.

22d, nine A.M.—Two stools, natural.

Diet: Tapioca, boiled milk, crackers, and chicken soup.

24th.—Diet of the house. 25th.—Discharged well.

CASE IV.—Josiah Phillips, aged eighteen years, seaman.

Symptoms: Has twenty slimy stools in twenty-four hours; tormina and tenesmus; some pain upon pressure on abdomen; tongue red at edges, and slightly furred in the centre; skin hot and dry; pulse 120.

Treatment: V.S. ℥xij. Ol. Ricini ℥i. statim. Pil. Cœrulæ grs. iij. at night.—Diet restricted to slippery elm tea and gum water.

18th, nine A.M.—Much better. Pulse more natural; skin relaxed; little pain in bowels; four stools since oil operated, these with much less pain.—Continue diet.

Seven P.M.—Two stools, these more natural.

Diet as before.—Pil. Cœrulæ gr. iij. at bedtime.

19th, nine A.M.—One stool, without pain, nearly natural; pulse seventy-five; skin pleasant; tongue cleaner in centre.

Continue diet, and in addition allowed tapioca and one cracker.

20th, nine A.M.—One stool.

Continue diet as yesterday.—Pil. Cœrulæ gr. iij. at bedtime.

21st, nine A.M —One stool, natural.

Diet: Tapioca, boiled milk, crackers, and chicken soup.

24th.—Diet of the house. 26th.—Discharged well.

